# Comparative Effectiveness of Pelvic Floor Muscle Training and Pharmacological Therapy in Women With Urinary Incontinence: An Observational Study

**DOI:** 10.7759/cureus.109488

**Published:** 2026-05-23

**Authors:** Manal A Alqahtani, Hind M Faqeeh, Gofran M Albraheem, Kholoud A Assiri, Jawaher H Al-lugbi, Mohammed A Alqarni, Husain Shamsan, Salman M AlNasheet

**Affiliations:** 1 Obstetrics and Gynecology, Khamis Mushayt Maternity and Children Hospital, Abha, SAU; 2 Obstetrics and Gynecology, AlKhamis Maternity and Children Hospital, Khamis Mushait, SAU; 3 Obstetrics and Gynecology, Al-Ahsaa Maternity and Children's Hospital, Al-Ahsaa, SAU; 4 Obstetrics and Gynecology, Armed Forces Hospital Southern Region, Khamis Mushait, SAU; 5 College of Medicine, I.M. Sechenov First Moscow State Medical University, Moscow, RUS; 6 Emergency Medicine, University Hospital of Wales, Cardiff, GBR

**Keywords:** antimuscarinic agents, beta-3 adrenergic agonists, female urinary incontinence, mixed urinary incontinence, pelvic floor muscle training, pharmacological therapy, stress urinary incontinence, urge urinary incontinence, urinary incontinence

## Abstract

Background

Urinary incontinence is a common and underreported condition in women that significantly impairs quality of life. Pelvic floor muscle training (PFMT) and pharmacological therapy are both widely used treatment options, but comparative real-world evidence on their effectiveness and tolerability remains limited, particularly in routine clinical practice settings.

Methods

This prospective comparative observational study included women aged ≥18 years with clinically diagnosed stress, urge, or mixed urinary incontinence attending outpatient clinics in Saudi Arabia. Participants were allocated to either PFMT or pharmacological therapy based on routine clinical decision-making. Baseline demographic and clinical characteristics were recorded, including urinary incontinence severity assessed using the International Consultation on Incontinence Questionnaire-Urinary Incontinence Short Form (ICIQ-UI SF). Outcomes were assessed after a treatment period of approximately 10-12 weeks. The primary outcome was the change in ICIQ-UI SF score. Secondary outcomes included reduction in weekly incontinence episodes, pad usage, patient-reported global improvement, achievement of ≥50% symptom reduction, complete continence, and adverse events.

Results

A total of 102 women were included (PFMT: n=52; pharmacotherapy: n=50). Baseline characteristics were comparable between groups. Both groups demonstrated improvement in urinary incontinence symptoms. The reduction in ICIQ-UI SF score was greater in the PFMT group compared with the pharmacotherapy group (-7.2 ± 3.1 vs -6.1 ± 3.4; p=0.048). Reduction in weekly incontinence episodes showed a non-significant trend favoring PFMT (-5.8 ± 3.0 vs -4.6 ± 3.3; p=0.06). Clinical improvement of ≥50% was observed in 38/52 (73.1%) in the PFMT group and 32/50 (64.0%) in the pharmacotherapy group. Complete continence was achieved in 11/52 (21.2%) and 8/50 (16.0%), respectively. Adverse events were significantly more frequent in the pharmacotherapy group (14/50 (28.0%) vs 3/52 (5.8%); p<0.01).

Conclusions

Both PFMT and pharmacological therapy were effective in improving urinary incontinence symptoms in women. PFMT demonstrated slightly greater improvement in symptom severity and a significantly lower rate of adverse effects. These findings support PFMT as a preferred first-line treatment option in routine clinical practice, while pharmacological therapy remains an important alternative for selected patients.

## Introduction

Urinary incontinence is a highly prevalent and often underreported condition that significantly affects quality of life among women worldwide [1-3]. It is characterized by involuntary leakage of urine and is commonly classified into stress, urge, and mixed subtypes [2,4]. Stress urinary incontinence results from impaired urethral support and pelvic floor dysfunction, leading to leakage during activities that increase intra-abdominal pressure [1,3]. Urge incontinence is primarily associated with detrusor overactivity, while mixed incontinence includes features of both mechanisms [1-5]. Although not life-threatening, urinary incontinence is associated with substantial physical, psychological, and social burden, including reduced self-esteem, social isolation, and increased risk of depression [3,4].

The prevalence of urinary incontinence increases with age, parity, obesity, and menopausal status. It is estimated that a substantial proportion of adult women experience some degree of symptoms, yet many do not seek medical care due to stigma or the misconception that it is a normal part of aging [1,6]. This contributes to underdiagnosis and undertreatment, particularly in primary care settings.

Management of urinary incontinence typically follows a stepwise approach, beginning with conservative measures. Pelvic floor muscle training (PFMT) is widely recommended as first-line therapy for stress and mixed urinary incontinence [2-6]. PFMT aims to strengthen the pelvic floor musculature, improve urethral support, and enhance voluntary continence mechanisms. Multiple studies have demonstrated its effectiveness in reducing symptom severity and improving quality of life, particularly when performed consistently and under supervision [5-10].

Pharmacological therapy is commonly used, particularly for urge-predominant symptoms. Antimuscarinic agents and beta-3 adrenergic agonists act by reducing detrusor overactivity and increasing bladder storage capacity [10,11]. While these agents are effective in reducing urgency and frequency symptoms, their use is often limited by adverse effects such as dry mouth, constipation, and blurred vision, which can negatively impact adherence and long-term persistence [11-13].

Despite the availability of both PFMT and pharmacological therapies, there remains variability in clinical practice regarding first-line treatment selection, particularly in real-world settings where patient adherence, tolerability, and resource availability influence outcomes [3,6]. Direct comparisons between these two treatment modalities in routine clinical populations remain limited, particularly in Middle Eastern populations, where cultural and healthcare system factors may influence treatment adherence and reporting behavior.

Given this context, there is a need for comparative evidence evaluating the effectiveness and tolerability of PFMT versus pharmacological therapy in women with urinary incontinence in real-world clinical practice. This study aims to address this gap by comparing clinical outcomes, symptom improvement, and adverse event profiles between these two commonly used treatment strategies.

## Materials and methods

Study design and setting

This was a comparative observational study conducted among women with urinary incontinence attending outpatient clinics as part of a residency research project in Saudi Arabia. The study compared outcomes between PFMT and pharmacological therapy in routine clinical practice. Participants were followed prospectively from initiation of treatment to a predefined follow-up period to assess symptom improvement and safety outcomes.

Study population

Women were eligible for inclusion if they were aged 18 years or older and had a clinical diagnosis of urinary incontinence (stress, urge, or mixed type) confirmed through history and clinical evaluation. Participants were required to have at least moderate symptom severity at baseline, defined using the International Consultation on Incontinence Questionnaire-Urinary Incontinence Short Form (ICIQ-UI SF) [14,15]. Exclusion criteria included pregnancy, previous surgical intervention for urinary incontinence within the last 12 months, neurological disorders affecting bladder function (such as multiple sclerosis or spinal cord injury), active urinary tract infection at baseline, or inability to complete follow-up assessments (Table 1).

**Table 1:** Inclusion and exclusion criteria for participant selection Data are presented as predefined eligibility criteria used for enrollment in this comparative observational study of women with urinary incontinence. ICIQ-UI SF denotes International Consultation on Incontinence Questionnaire–Urinary Incontinence Short Form. Participants were required to meet all inclusion criteria and none of the exclusion criteria before enrollment.

Inclusion Criteria	Exclusion Criteria
Women aged 18 years or older	Pregnancy
Clinical diagnosis of stress, urge, or mixed urinary incontinence	Previous surgical intervention for urinary incontinence within the last 12 months
Moderate or greater symptom severity at baseline based on the ICIQ-UI SF	Neurological disorders affecting bladder function (e.g., multiple sclerosis or spinal cord injury)
Ability to participate in follow-up assessments	Active urinary tract infection at baseline
Provision of informed consent	Inability to complete follow-up assessments

Study groups and interventions

Participants were allocated into two treatment groups based on clinical decision-making in routine care. The first group received PFMT, which included instruction in structured pelvic floor exercises, either supervised by a trained clinician or provided as a standardized home-based program. Participants were advised to perform exercises regularly over a treatment period of approximately 10-12 weeks. The second group received pharmacological therapy, which included either antimuscarinic agents or beta-3 adrenergic agonists prescribed according to clinician preference and patient suitability. Medication selection, dosing, and adjustments were guided by standard clinical practice. Treatment adherence was monitored through patient self-reporting and follow-up clinic visits.

Data collection and study variables

Baseline data were collected at enrollment and included demographic characteristics (age, body mass index, menopausal status, parity, and smoking status), as well as clinical characteristics such as type and duration of urinary incontinence. Severity of symptoms was assessed using the ICIQ-UI SF score, along with the number of incontinence episodes per week and daily pad usage. Treatment-related variables included the type of intervention, duration of therapy, and adherence level. Follow-up assessments were performed after completion of the treatment period and included repeat measurement of symptom severity, frequency of incontinence episodes, pad usage, and patient-reported global improvement. Adverse events were recorded in both groups, particularly anticholinergic side effects in the pharmacotherapy group and any discomfort or musculoskeletal complaints in the PFMT group.

Outcomes

The primary outcome was the change in urinary incontinence severity, measured by the difference in ICIQ-UI SF score from baseline to follow-up. Secondary outcomes included reduction in weekly incontinence episodes, change in pad usage, patient-reported global improvement, achievement of ≥50% symptom reduction, complete continence at follow-up, and incidence of adverse effects.

Statistical analysis

Continuous variables were summarized using means and standard deviations or medians and interquartile ranges, depending on distribution. Categorical variables were presented as counts and percentages. Comparisons between the PFMT and pharmacotherapy groups were performed using independent samples t-tests or Mann-Whitney U tests for continuous variables, and chi-square tests or Fisher’s exact tests for categorical variables, as appropriate. A two-sided p-value of less than 0.05 was considered statistically significant. Statistical analyses were conducted using standard statistical software. No imputation was performed for missing data; analyses were based on available cases.

Ethical considerations

The study was conducted in accordance with the principles of the Declaration of Helsinki. Institutional approval was obtained before data collection. The Ethics Committee of the Ministry of Health, Saudi Arabia, issued approval REC-2022-14225. All participants provided informed consent before enrollment. Patient confidentiality was maintained throughout the study, and all data were anonymized before analysis.

## Results

Baseline characteristics of the study population

A total of 102 women with urinary incontinence were included in the analysis, with 52 assigned to pelvic floor muscle training (PFMT) and 50 to pharmacological therapy. The mean age was similar between groups (PFMT: 54.3 ± 10.8 years; pharmacotherapy: 56.1 ± 11.2 years; p = 0.42). Mean BMI was also comparable (29.6 ± 4.8 vs 30.2 ± 5.1 kg/m²; p = 0.58). Most participants in both groups were postmenopausal (36/52 (69.2%) in PFMT vs 37/50 (74.0%) in pharmacotherapy; p = 0.61). Parity distribution was similar between groups (median 4 (IQR 2-6) vs 4 (IQR 3-6); p = 0.77), and smoking prevalence was low and comparable (6/52 (11.5%) vs 5/50 (10.0%); p = 0.81). The distribution of incontinence type did not differ significantly between groups (χ²(2) = 0.13, p = 0.94, Cramér’s V = 0.04), with stress incontinence observed in 21/52 (40.4%) vs 19/50 (38.0%), urge incontinence in 12/52 (23.1%) vs 11/50 (22.0%), and mixed incontinence in 19/52 (36.5%) vs 20/50 (40.0%) in the PFMT and pharmacotherapy groups, respectively. Postmenopausal status (χ²(1) = 0.28, p = 0.59, Cramér’s V = 0.05) and smoking prevalence (χ²(1) = 0.06, p = 0.80, Cramér’s V = 0.02) were also comparable between groups. These findings are summarized in Table 2.

**Table 2:** Baseline characteristics of women with urinary incontinence according to treatment group Data are presented as mean ± standard deviation, median (interquartile range), or number (percentage), unless otherwise specified. Percentages may not sum to 100 due to rounding. Comparisons between groups were performed using independent t-tests, Mann-Whitney U tests, or Chi-square tests as appropriate. For categorical variables analyzed using chi-square tests, corresponding χ² statistics, degrees of freedom, and effect sizes (Cramér’s V) are reported in the Results section. No statistically significant differences were observed between groups at baseline. PFMT, pelvic floor muscle training; BMI, body mass index.

Characteristic		PFMT Group (n=52)	Pharmacotherapy Group (n=50)	p-value
Age (years), mean ± SD		54.3 ± 10.8	56.1 ± 11.2	0.42
BMI (kg/m²), mean ± SD		29.6 ± 4.8	30.2 ± 5.1	0.58
Postmenopausal, n (%)		36 (69.2)	37 (74.0)	0.61
Parity, median (IQR)		4 (2–6)	4 (3–6)	0.77
Smoking, n (%)		6 (11.5)	5 (10.0)	0.81
Type of incontinence, n (%)	Stress	21 (40.4)	19 (38.0)	0.84
	Urge	12 (23.1)	11 (22.0)	
	Mixed	19 (36.5)	20 (40.0)	

Baseline severity of urinary incontinence and symptom burden

At baseline, the severity of urinary incontinence was comparable between groups. The mean ICIQ-UI Short Form score was 15.8 ± 3.9 in the PFMT group and 16.1 ± 4.1 in the pharmacotherapy group (p = 0.71). Median leakage episodes were 9 (IQR 6-14) per week in the PFMT group compared with 10 (IQR 7-15) in the pharmacotherapy group (p = 0.39). Pad usage was similar, with a median of 2 (IQR 1-3) pads/day in the PFMT group and 2 (IQR 1-4) in the pharmacotherapy group (p = 0.64). The mean duration of symptoms was 3.8 ± 2.1 years in the PFMT group and 4.1 ± 2.4 years in the pharmacotherapy group (p = 0.53). These baseline severity measures are presented in Table 3.

**Table 3:** Baseline severity of urinary incontinence and symptom burden Data are presented as mean ± standard deviation or median (interquartile range). Baseline symptom severity, frequency of leakage episodes, pad usage, and symptom duration were comparable between treatment groups, with no statistically significant differences observed. PFMT, pelvic floor muscle training; ICIQ-UI SF, International Consultation on Incontinence Questionnaire–Urinary Incontinence Short Form.

Variable	PFMT Group	Pharmacotherapy Group	p-value
ICIQ-UI SF score, mean ± SD	15.8 ± 3.9	16.1 ± 4.1	0.71
Leakage episodes/week, median (IQR)	9 (6–14)	10 (7–15)	0.39
Pads/day, median (IQR)	2 (1–3)	2 (1–4)	0.64
Duration of symptoms (years), mean ± SD	3.8 ± 2.1	4.1 ± 2.4	0.53

Treatment characteristics and adherence

Treatment exposure characteristics are shown in Table 4. In the PFMT group, 34/52 (65.4%) of participants underwent supervised training, with a median of 3 (IQR 2-4) sessions per week. The mean treatment duration was 10.2 ± 3.6 weeks in the PFMT group and 10.5 ± 3.2 weeks in the pharmacotherapy group. In the pharmacological therapy group, 31/50 (62.0%) received antimuscarinic agents and 19/50 (38.0%) received a beta-3 agonist. Medication adherence ≥80% was reported in 38/50 (76.0%) of participants. PFMT adherence was rated as good in 29/52 (55.8%) of participants.

**Table 4:** Treatment characteristics and adherence in pelvic floor muscle training and pharmacotherapy groups Data are presented as number (percentage), mean ± standard deviation, or median (interquartile range). Antimuscarinic agents included oxybutynin, solifenacin, or equivalent agents, while beta-3 agonist therapy primarily included mirabegron. Medication adherence was defined as self-reported intake of ≥80% of prescribed doses. PFMT adherence was categorized based on patient-reported compliance with prescribed exercise frequency. PFMT, pelvic floor muscle training.

Variable		PFMT Group	Pharmacotherapy Group
Supervised PFMT, n (%)		34 (65.4)	—
Weekly PFMT sessions, median (IQR)		3 (2–4)	—
Treatment duration (weeks), mean ± SD		10.2 ± 3.6	10.5 ± 3.2
Drug type, n (%)	Antimuscarinic	—	31 (62.0)
	Beta-3 agonist	—	19 (38.0)
Medication adherence ≥80%, n (%)		—	38 (76.0)
PFMT adherence good, n (%)		29 (55.8)	—

Primary and secondary clinical outcomes

At follow-up, both groups demonstrated improvement in symptoms; however, the magnitude of improvement differed slightly between groups (Table 5). The mean reduction in ICIQ-UI score was -7.2 ± 3.1 in the PFMT group compared with -6.1 ± 3.4 in the pharmacotherapy group (p = 0.048). The reduction in weekly incontinence episodes was -5.8 ± 3.0 in the PFMT group and -4.6 ± 3.3 in the pharmacotherapy group (p = 0.06). Reduction in pad usage was similar between groups (-1.3 ± 0.8 vs -1.1 ± 0.9 pads/day; p = 0.21). Patient-reported global improvement did not differ significantly between groups (χ²(2) = 1.08, p = 0.58, Cramér’s V = 0.10). Significant improvement was reported by 32/52 (61.5%) in the PFMT group and 27/50 (54.0%) in the pharmacotherapy group, while 15/52 (28.8%) vs 15/50 (30.0%) reported moderate improvement, and 5/52 (9.7%) vs 8/50 (16.0%) reported minimal or no improvement.

**Table 5:** Primary and secondary clinical outcomes at follow-up Data are presented as mean ± standard deviation or percentage of participants. The primary outcome was the change in ICIQ-UI SF score from baseline to follow-up. Secondary outcomes included reduction in weekly incontinence episodes, change in pad usage, and patient-reported global improvement categorized as significant, moderate, or minimal/no improvement. Statistical comparisons were performed using appropriate parametric, non-parametric, or chi-square tests as indicated. Corresponding chi-square statistics, degrees of freedom, and effect sizes are reported in the Results section. PFMT, pelvic floor muscle training; ICIQ-UI SF, International Consultation on Incontinence Questionnaire–Urinary Incontinence Short Form.

Outcome		PFMT Group	Pharmacotherapy Group	p-value
Change in ICIQ score		-7.2 ± 3.1	-6.1 ± 3.4	0.048
Reduction in episodes/week		-5.8 ± 3.0	-4.6 ± 3.3	0.06
Pad reduction (pads/day)		-1.3 ± 0.8	-1.1 ± 0.9	0.21
Patient-reported improvement (%)	Significant	61.5	54.0	0.31
	Moderate	28.8	30.0	
	Minimal/none	9.7	16.0	

Clinical response, cure rates, and adverse events

Clinical response outcomes and safety data are presented in Table 6. A ≥50% reduction in symptoms was achieved in 38/52 (73.1%) of participants in the PFMT group compared with 32/50 (64.0%) in the pharmacotherapy group, with no statistically significant difference between groups (χ²(1) = 0.98, p = 0.32, Cramér’s V = 0.10). Complete continence at follow-up was observed in 11/52 (21.2%) vs 8/50 (16.0%), respectively (χ²(1) = 0.45, p = 0.50, Cramér’s V = 0.07). Adverse effects were significantly more common in the pharmacotherapy group, occurring in 14/50 (28.0%) compared with 3/52 (5.8%) in the PFMT group (χ²(1) = 9.07, p = 0.003, Cramér’s V = 0.30). Reported side effects in the pharmacotherapy group included dry mouth in 6/50 (12.0%), constipation in 4/50 (8.0%), and blurred vision in 2/50 (4.0%). The dropout rate was low and comparable between groups (4/52 [7.7%] vs 6/50 [12.0%]; χ²(1) = 0.53, p = 0.46, Cramér’s V = 0.07).

**Table 6:** Clinical response, continence outcomes, and adverse events Data are presented as numbers (percentages) unless otherwise specified. Clinical improvement was defined as ≥50% reduction in baseline symptom burden. Complete continence was defined as no reported urinary leakage during the follow-up period. Adverse events were actively recorded during follow-up visits and included treatment-specific side effects. Comparisons between groups were performed using chi-square or Fisher’s exact tests as appropriate. Corresponding Chi-square statistics, degrees of freedom, and effect sizes (Cramér’s V) are provided in the Results section. PFMT, pelvic floor muscle training.

Outcome	PFMT Group	Pharmacotherapy Group	p-value
Clinical improvement ≥50% reduction in symptoms	38 (73.1)	32 (64.0)	0.32
Complete continence at follow-up	11 (21.2)	8 (16.0)	0.48
Any adverse effects	3 (5.8)	14 (28.0)	<0.01
Dry mouth	—	6 (12.0)	
Constipation	—	4 (8.0)	
Blurred vision	—	2 (4.0)	
Dropout rate	4 (7.7)	6 (12.0)	0.41

## Discussion

In this comparative study of women with urinary incontinence, both PFMT and pharmacological therapy were associated with clinically meaningful improvement in symptoms. However, PFMT demonstrated a modest but statistically significantly greater reduction in ICIQ-UI SF scores compared with pharmacological therapy (-7.2 ± 3.1 vs -6.1 ± 3.4; p = 0.048). Although reductions in weekly incontinence episodes favored PFMT, this difference did not reach statistical significance. Importantly, pharmacological therapy was associated with a substantially higher rate of adverse effects (14/50 (28.0%) vs 3/52 (5.8%); p < 0.01). These findings suggest that both interventions are effective, but PFMT may offer a more favorable balance between efficacy and tolerability in routine clinical practice.

The observed improvement in both groups aligns with the established effectiveness of conservative and pharmacological approaches for urinary incontinence. PFMT likely improves continence by strengthening pelvic floor musculature, enhancing urethral support, and improving voluntary control during increases in intra-abdominal pressure. The greater reduction in symptom severity scores observed in the PFMT group may reflect sustained neuromuscular adaptation over the treatment period, particularly in participants with good adherence [5-8].

Pharmacological therapy, primarily involving antimuscarinic agents and beta-3 agonists, targets detrusor overactivity and reduces urgency symptoms. While this mechanism is effective, the slightly smaller improvement in symptom scores compared with PFMT in this cohort may be influenced by variability in drug response, adherence limitations, and the burden of side effects, which are known to reduce persistence with therapy [16-18].

The findings of this study are broadly consistent with prior evidence demonstrating that PFMT is a first-line therapy for stress and mixed urinary incontinence, with effectiveness comparable to or exceeding pharmacological therapy in some populations. Randomized trials have shown that structured PFMT programs significantly reduce leakage episodes and improve quality of life scores, particularly when supervised and supported by adherence reinforcement strategies [5-9].

Similarly, pharmacological therapy has been shown to provide symptomatic relief, especially in urge-predominant incontinence; however, real-world effectiveness is often limited by discontinuation due to adverse effects such as dry mouth, constipation, and blurred vision. The higher adverse event rate observed in this study (28.0%) is consistent with previously reported discontinuation rates in antimuscarinic therapy cohorts.

These findings have practical implications for first-line management of urinary incontinence in women. PFMT should remain a cornerstone of initial therapy, particularly given its favorable safety profile and comparable or superior efficacy in symptom reduction. The significantly lower adverse event burden observed in the PFMT group supports its use as a preferred initial intervention, especially in younger patients or those with concerns regarding medication tolerance.

Pharmacological therapy remains an important option, particularly for patients with predominant urge symptoms or those who do not respond adequately to conservative measures. However, careful patient counseling regarding potential side effects and adherence challenges is essential to optimize outcomes.

Limitations

Several limitations should be acknowledged. First, the non-randomized design introduces the possibility of selection bias, as treatment allocation was based on clinical decision-making rather than random assignment. Second, adherence was assessed using self-reported measures, which may be subject to reporting bias. Third, the follow-up duration was relatively short, limiting conclusions regarding the long-term durability of treatment effects. Fourth, the sample size, while adequate for detecting differences in primary outcomes, may not have been powered to identify smaller differences in secondary endpoints such as continence rates. Additionally, subgroup analyses by incontinence type were limited by sample size and were not formally powered, which restricts the interpretation of differential treatment effects across stress, urge, and mixed subtypes.

## Conclusions

In this comparative study of women with urinary incontinence, both PFMT and pharmacological therapy were associated with significant improvement in symptoms over the follow-up period. PFMT demonstrated a modest but statistically greater reduction in symptom severity compared with pharmacological therapy, along with a consistently lower rate of adverse effects. Although both interventions were clinically effective, the safety and tolerability profile of PFMT appeared more favorable in this cohort, supporting its role as a cornerstone of conservative management.

These findings reinforce current guideline recommendations that prioritize PFMT as first-line therapy for female urinary incontinence. Pharmacological therapy remains an important treatment option, particularly for patients with urge-predominant symptoms or inadequate response to conservative measures; however, its use should be balanced against the potential for adverse effects and reduced adherence. Further randomized studies with longer follow-up are warranted to better define long-term comparative effectiveness and to optimize patient selection for each therapeutic approach.
